# Fulminant lupus pneumonitis complicating systemic lupus erythematosus in the elderly

**DOI:** 10.1002/ccr3.6966

**Published:** 2023-02-23

**Authors:** Wiem Helali, Mounira El Euch, Cyrine Sassi, Asma Kefi, Fethi Ben Hamida, Khaoula Ben Abdelghani, Sami Turki, Ezzedine Abderrahim

**Affiliations:** ^1^ Internal Medicine Department A Charles Nicolle hospital of Tunis Tunis Tunisia; ^2^ University of Tunis Manar Tunis Tunisia; ^3^ Research Laboratory of Kidney Diseases (LR00SP01) Charles Nicolle Hospital Tunis Tunisia

**Keywords:** elderly, pneumonitis, lupus

## Abstract

Fulminant lupus pneumonitis is a rare complication of SLE. We report a case of 75 years‐old male patient with SLE who developed pneumonia and severe respiratory failure requiring mechanical ventilation. Refractory respiratory distress complicating noninfectious fulminant lupus pneumonitis did not respond to methylprednisolone and intravenous immunoglobulin treatment.

## Abstract 2

FLP in SLE of the elderly is a rare and severe condition. Each practitioner has to focus on respiratory failure, which is severe and life‐threatening.

## 1 BACKGROUND

Systemic lupus erythematosus (SLE) is a frequent connective tissue disease that can involve several organs such as skin, kidneys, lungs, brain, and heart.^1^ Although commonly seen in young women, it can affect men as well as elderly patients.^2^ Pleuropulmonary manifestations are frequent in SLE, particularly pleuritic, and older age is among the factors associated with pulmonary involvement.^3^ To date, few cases of non‐infectious fulminant lupus pneumonitis (FLP) have been reported in the literature and concerned young patients. We hereby describe the first case of SLE complication by FLP in an elderly patient.

## 2 OBSERVATION

A 75‐year‐old male presented with a history of weakness for a few months. He had a familial history of SLE (his daughter). On general examination, the patient was conscious, with stable cardio‐respiratory vitals. The joint examination was normal and pallor was present. His lab stix found proteinuria. Diagnosis of SLE was retained since the presence of 4 criteria of ACR was detected: kidney involvement with proteinuria (3.27 g/24 h), autoimmune hemolytic anemia (7.3 g/dL), thrombocytopenia (96,000 elts/mm^3^), and positivity of anti‐nuclear antibodies (title = 1/400) and anti‐double stranded DNA antibodies. He was treated with 3 pulses of methylprednisolone (1 g/day) relayed by oral prednisone at 1 mg/kg/day in combination with cyclophosphamide. Initial evolution was favorable with clinical and biological improvement (Hb = 11 g/dL and proteinuria at 1 g/24 h). However, a month later, he presented acute dyspnea. On examination, he had no fever, tachycardia (HR 100/min), and tachypnoea at 24/min. On auscultation, loud crackles were audible over both lungs. Biological analyzes were normal (C‐reactive protein = 15 mg/L, Procalcitonin = 0,73; WBC = 4200 cells/mm^3^). Blood arterial gases revealed severe respiratory failure with hypoxemia (pO_2_ 39.4 mmHg, pCO_2_ 30 mmHg, Sat 75%). There were negative repeated sputum and blood cultures. Bronchoalveolar lavage (BAL) cultures were also negative. The Golde score was under 100. Chest X‐ray revealed a bilateral alveolar syndrome. High‐resolution computed chest tomography showed bilateral ground glass opacities. Tests for HIV, CMV, EBV, and viral C, and B hepatitis were negative. Sputum examination for acid‐fast bacillus was negative. Although infectious pneumopathy was not very probable, we started empirical antibiotics (ciprofloxacin and spiramycin) with noninvasive ventilation and steroids (a pulse of intravenous methylprednisolone). Repeated blood gases evaluations showed no improvement and in two days, the patient progressed into severe respiratory failure. He required mechanical ventilation on volume support mode and we added intravenous immunoglobulin. Unfortunately, our patient deceased from refractory respiratory distress.

## 3 DISCUSSION

Herein, we report a unique case of FLP in an elderly patient.

Pulmonary manifestations in SLE represent one of the most common involvements and are prognostic for the patient's outcome.^4^ In the study of Hsu and al, the most common cause of admission of patients with SLE in the intensive care unit was pneumonia and acute respiratory distress syndrome, with a mortality rate of 47%.^5^

FLP is a rare and highly fatal complication of SLE, with up to a 50% mortality rate.^1^ Only a few cases of non‐infectious FLP have been reported in the literature, affecting young patients with SLE.^1^, ^6^, ^7^, ^8^ Similarly to our patient, FLP tends to affect patients with a recent diagnosis of SLE and its clinical features often mimic atypical pneumonia. The diagnosis of FLP can be challenging, as it is made based on a combination of factors such as recently diagnosed SLE, imaging results, and negative results from infectious investigation. It is important to note that while lupus may increase the risk of fatal adenoviral infections,^9^ none of the reported cases in the literature has evaluated patients for this possibility.

FLP is a serious disease requiring multi‐disciplinary cooperation to minimalize the risk of fatal outcomes.^10^ A combination of high‐dose corticosteroids including bolus intravenous methylprednisolone with respiratory support, when needed, is still the gold standard of drug therapy for FLP, without any validated controlled trials.^11^ Although our patient did not respond to treatment, intravenous pulse of cyclophosphamide combined with methylprednisolone proved its success in other similar cases.^10^, ^12^ Intravenous immunoglobulin therapy, prescribed without success in our patient, is generally limited for patients with SLE and resistant pleural effusion. Plasmapheresis, usually reserved for refractory cases of lupus pneumonitis late in the course, can be useful in the treatment of selected cases of FLP.^7^, ^13^ In our patient's case, urgent plasma exchange could not be provided.

## 4 CONCLUSION

FLP is an uncommon yet severe syndrome with a high rate of mortality. The diagnosis can be a real challenge, especially when FLP is the initial presentation of SLE. The treatment in an intensive care unit is urgent and requires a combination of intensive immunosuppressive drugs with steroids. Raising awareness among clinicians about this rare complication, along with a close cooperation between different medical fields, are of key importance if we are to provide an early and better management.

## AUTHOR CONTRIBUTIONS

MEE conceived the study and wrote the paper. WH helped in writing the paper. AK performed the experiments. CS analyzed the data. FBH and ST contributed to the management of the patient. KBA financed the project. ST and EA supervised and approbated the project.

## FUNDING INFORMATION

This research did not receive any specific grant from any funding agency in the public, commercial, or not‐for‐profit sector.

## CONFLICT OF INTEREST STATEMENT

The authors do not declare any conflict of interest.

## ETHICAL STATEMENT

Not available (the patient deceased).

## CONSENT

Written informed consent was not obtained from the patient because of his death.

## Data Availability

Clinical, biological, and radiological data are available on medical file of the patient in internal medicine department.
